# Turkish Version of the Cognitive Distortions Questionnaire: Psychometric Properties

**DOI:** 10.1155/2015/694853

**Published:** 2015-08-13

**Authors:** Sedat Batmaz, Sibel Kocbiyik, Ozgur Ahmet Yuncu

**Affiliations:** ^1^Department of Psychiatry, School of Medicine, Gaziosmanpasa University, 60000 Tokat, Turkey; ^2^Psychiatry Clinic, Ataturk Training and Research Hospital, 06000 Ankara, Turkey; ^3^Psychiatry Clinic, Ankara Training and Research Hospital, 06000 Ankara, Turkey

## Abstract

Cognitive distortions are interrelated with all layers of cognitions, and they may be part of the treatment once they are accessed, identified, labeled, and changed. From both a research and a clinical perspective, it is of utmost importance to disentangle cognitive distortions from similar constructs. Recently, the Cognitive Distortions Questionnaire (CD-Quest), a brief and comprehensive measure, was developed to assess both the frequency and the intensity of cognitive distortions. The aim of the present study was to assess the psychometric properties of the Turkish version of the CD-Quest in a psychiatric outpatient sample. Demographic and clinical data of the participants were analyzed by descriptive statistics. For group comparisons, Student's *t*-test was applied. An exploratory principal components factor analysis was performed, followed by an oblique rotation. To assess the internal consistency of the scale Cronbach's *α* was computed. The correlation coefficient was calculated for test-retest reliability over a 4-week period. For concurrent validity, bivariate Pearson correlation analyses were conducted with the measures of mood severity and negatively biased cognitions. The results revealed that the scale had excellent internal consistency, good test-retest reliability, unidimensional factor structure, and evidence of concurrent and discriminant validity.

## 1. Introduction

According to the cognitive model of depression proposed by Beck [1, 2], the negative cognitive triad (i.e., rigid negative views of the self, others and the world, and the future) plays a central role in the development and maintenance of the disorder. This triad may manifest itself as negative automatic thoughts, and these may indeed be the byproducts of the underlying dysfunctional beliefs (i.e., dysfunctional attitudes, personal rules, and conditional assumptions) and schemata (i.e., core beliefs) [1, 2]. This traditionally defined three-layer structure of negative cognitions is theoretically thought to be related to the etiology, maintenance, and treatment of mental disorders, including depression. Cognitive distortions, or thinking errors, are also primarily interrelated with these three layers of cognitions, and they may be part of the treatment once they are accessed, identified, labeled, and changed by the therapist in cooperation with the patient during psychotherapy sessions [3].

A cognitive distortion has been described as “a cognitive process that does not consist of content, [and] contribute[s] to the transformation of dysfunctional attitudes and environmental events into automatic negative thoughts” [4]. Beck initially defined six types of cognitive distortions, that is, arbitrary inference, selective abstraction, overgeneralization, magnification/minimization, personalization, and absolutistic dichotomous thinking [1], to which Burns later added should statements, disqualifying the positive, emotional reasoning, and labeling and mislabeling. He also renamed some of the originally named distortions, for example, jumping to conclusions (mind reading and fortune telling), mental filter, and all-or-nothing thinking [5]. From both a research and a clinical perspective, it is of utmost importance to disentangle cognitive distortions from the above listed similar constructs, since cognitive distortions “may include logical errors, but in most cases it is the evaluation of the information itself that is aberrant, for instance by ascribing an unwarranted negative (or positive) implication to the meaning of information. The resulting evaluation is often deleterious to how the patient subsequently perceives, thinks, feels, plans, and/or behaves, and may lead to maladaptive coping” [6]. What the authors actually refer to by “similar constructs” may be summarized as follows. (a) Negative automatic thoughts (NAT): these are conscious, repetitive, automatic, and biased thoughts, which thematically include negative content about the self, the world, and the future [1–3]. NATs are different from cognitive distortions in that not all NATs are sufficiently negative to a point where they may be called distortions [6]. Both NATs and cognitive distortions are evaluated in therapy to identify underlying schemas or dysfunctional attitudes [3]. (b) Intermediate beliefs: these thoughts represent “deeper, often unarticulated ideas or understandings that patients have about themselves, others, and their personal worlds, and give rise to specific automatic thoughts” [3]. They consist of personal rules, dysfunctional attitudes, and biased assumptions. Generally their misinterpretation results in cognitive errors. (c) Core beliefs: these are rigid, global, persistent ideas about oneself, others, or the world. Core beliefs may only be identified with a thorough questioning of the patient using specific cognitive techniques, for example, the downward arrow [3]. Core beliefs also tend to give rise to cognitive distortions, but they are not distortions themselves.

Although there are psychometrically sound scales for the assessment of these similar cognitive constructs, that is, negative automatic thoughts [7–9] and the deeper schemata [10–12], surprisingly little research has focused specifically on the assessment of cognitive distortions. Although this may partly be explained by the confusion of what exactly is being referred to with these constructs [6, 13], it may also have to do with the paucity of valid instruments to undertake scientific research. The literature review has provided us with the following measures to assess cognitive distortions: (a) the Cognitive Error Questionnaire (CEQ) [14], (b) the Inventory of Cognitive Distortions (ICD) [15], (c) the Cognitive Distortions Scale (CDS) [16], (d) the Cognitive Bias Questionnaire (CBQ) [17], (e) the Cognitive Distortion Scales [18], and (f) the Cognitive Error Rating Scales (CERS) [6]. There has been varying amount of empirical support for the use of all of these measures in the literature [14, 15, 19–23]. Yet, in 2011, de Oliveira developed the Cognitive Distortions Questionnaire (CD-Quest), a briefer and more comprehensive measure with a user friendly scoring grid to assess both the frequency and intensity of cognitive distortions occurring in a broad range of clinical occasions rather than only focusing on either interpersonal/social or achievement situations [24, 25].

The CD-Quest consists of 15 items, all of which are rated to reflect both the frequency and the intensity of the respective cognitive distortions. The included cognitive distortions in the scale are as follows: (a) dichotomous thinking (all-or-nothing and black-or-white thinking), (b) fortune telling (catastrophizing), (c) discounting the positive, (d) emotional reasoning, (e) labeling, (f) magnification/minimization, (g) selective abstraction (mental filter, tunnel vision), (h) mind reading, (i) overgeneralization, (j) personalization, (k) should statements (“musts,” “ought tos,” and “have tos”), (l) jumping to conclusions (arbitrary inference), (m) blaming others or oneself, (n) “What if…?” statements, and (o) unfair comparisons. The only previously published psychometric study of the CD-Quest conducted in an undergraduate student sample demonstrated that the scale has good internal consistency (Cronbach's *α* = 0.85) and significant convergent validity with self-report measures of depression (*r* = 0.65) and anxiety (*r* = 0.52). It was also shown that the CD-Quest was able to differentiate depressed and anxious groups from participants without mood symptoms. The factor analysis revealed that a one-factor solution best fit the data [24]. On the other hand, the initial psychometric study of the CD-Quest had revealed a four-factor solution [25]. These four factors consisted of the following cognitive distortions: Factor I, dichotomous thinking, selective abstraction, personalizing, should statements, “What if…?” statements, and unfair comparisons; Factor II, emotional reasoning, labeling, mind reading, and jumping to conclusions; Factor III, fortune telling, discounting positives, and magnification/minimization; and Factor IV, overgeneralizing and blaming [25].

The aim of the present study was to assess the psychometric properties of the Turkish version of the CD-Quest in a psychiatric outpatient sample with mood symptoms. The hypotheses of the study were that the CD-Quest would correlate significantly with measures of mood symptoms, a similar scale measuring cognitive distortions, and measures of negatively biased cognitions and that the CD-Quest would be able to distinguish between clinical and nonclinical individuals.

## 2. Materials and Methods

### 2.1. Participants

For the present study, a total of 269 psychiatric outpatients with predominantly mood symptoms aged 18 and older presenting to three tertiary healthcare services in two different cities were recruited. Participants were excluded from the study if they (i) were diagnosed with psychotic disorders, bipolar mood disorders, organic mental disorders, dementia, and/or mental retardation, (ii) suffered from an uncontrolled medical/neurologic disorder, (iii) were suicidal at the time of the intake interview, and (iv) had a history of head trauma, recent brain surgery, or electroconvulsive therapy.

### 2.2. Measures

Demographic and clinical data form: this form was developed by the researchers and the demographic data, that is, age, gender, level of education, marital status, occupation status, and clinical variables, that is, diagnosis, were recorded onto it.

Mini International Neuropsychiatric Interview (MINI) [26]: the MINI is a structured clinical diagnostic interview for mental disorders. In the present study, all participants were diagnosed according to the* Diagnostic and Statistical Manual of Mental Disorders* version IV (DSM-IV) [27] with the Turkish version of the MINI [28].

Cognitive Distortions Questionnaire (CD-Quest) [24]: this is a self-report questionnaire used to assess the frequency and intensity of 15 types of cognitive distortions. The respondents are expected to rate their experiences with the explained and exemplified cognitive distortions over the previous week. The respondents are asked how often these cognitive distortions occurred and are given four choices: (a) never, (b) occasional (1-2 days), (c) most of the time (3–5 days), and (d) almost always (6-7 days). Similarly, for the intensity, the respondents are asked how much they believed in their cognitive distortions and are given four choices: (a) not at all, (b) a little (up to 30%), (c) much (30–70%), and (d) very much (more than 70%). The frequency and intensity responses are grouped together to form a four-by-four grid, and every cell in this grid is assigned a score ranging from 0 to 5. Therefore, from this questionnaire three different scores may be obtained: (i) frequency score, (ii) intensity score, and (iii) total (composite) score. The scores of each item in the questionnaire are summed together to yield a total (possible range = 0–75). For the present study, only the total scores were computed. The total score of the questionnaire has been reported to highly correlate with its subscales (*r* = 0.95 for frequency and *r* = 0.96 for intensity, both *p* values < 0.01) [25]. Therefore, given these correlation coefficients, these subscales may be regarded as measuring the same as the total score of the questionnaire, and computing only the total score would be sufficient to report. For the translation of the CD-Quest, guidelines widely used in cross-cultural research were followed [29, 30]. First, the developer of the scale was contacted by e-mail, and after his approval, the scale was translated into Turkish by the first author of this paper. The translated scale was independently back-translated by two bilingual experts in the field, and all translations were compared with the original scale. After reviewing the original and translated versions, a final version of consensus was adopted.

Cognitive Distortions Scale (CDS) [16]: this 20-item self-report scale was developed to assess 10 types of cognitive distortions, that is, mind reading, catastrophizing, all-or-nothing thinking, emotional reasoning, labeling, mental filter, overgeneralization, personalization, should statements, and minimizing the positive, in two specific situations, one related to the interpersonal (social) and one to the achievement domains. The scale was shown to exhibit good psychometric properties (Cronbach's *α*'s ranging from 0.75 to 0.85). For the present study, the Turkish version of the scale was used, which was reported to have excellent internal consistency (Cronbach's *α*'s ranging from 0.92 to 0.93), and significant correlations with measures of depression and anxiety severity, and negatively biased thinking [21]. Both the subscale scores (possible range = 10–70) and the total score of the CDS (possible range = 20–140) were used in the statistical analyses.

Automatic Thoughts Questionnaire, negative (ATQ) [7]: the ATQ is a 30-item 5-point Likert type self-report scale that assesses the frequency of negative automatic thoughts. For each item, respondents are asked to indicate how frequently each thought occurred during the past week (1 = not at all, 5 = all the time). It was reported to have excellent psychometric properties and to differentiate between depressed and nondepressed groups [31, 32]. The Turkish version of the ATQ, which was shown to exhibit good reliability (Cronbach's *α* = 0.93) and concurrent and discriminant validity, was used for the present study [33]. Only the total score of the ATQ was used in the analyses (possible range = 30–150).

Dysfunctional Attitudes Scale, Form A (DAS) [10]: the DAS is a 40-item 7-point Likert type self-report scale which assesses underlying dysfunctional assumptions about the need for approval and perfectionism. Each item of the DAS is rated to indicate how much the respondent agrees with the given statement (1 = totally disagree, 7 = totally agree). It was previously consistently shown that the DAS may reliably distinguish between clinical and nonclinical groups [34–37] and that it has sound psychometric properties [38–40]. The Turkish version of the DAS was also reported to be a reliable and valid tool [41, 42]. Recently, an abbreviated form of the Turkish version of the DAS (DAS-R) with similar reliability and validity results to the original scale was presented, and this version was used in this study [43]. The subscale scores for need for approval and perfectionism and the total score of the DAS-R (possible ranges 10–70; 8–56; 18–126, resp.) were computed for statistical analyses.

Hospital Anxiety and Depression Scale (HADS) [44]: the HADS consists of 14 items, equally divided into two subscales of depression and anxiety. It is a 4-point Likert type self-report instrument, and a cut-off score of 7 for depression and 10 for anxiety has been proposed. A recent review suggested that the HADS had a sensitivity of 70% and specificity of 83% for depression at this cut-off score [45]. The Turkish version of the HADS, which was used in the present study, was shown to be a reliable (Cronbach's *α* = 0.78 for depression and 0.85 for anxiety) and valid instrument [46]. Both subscale scores were calculated for analyses.

### 2.3. Procedure

The diagnostic interview was administered face to face at intake by trained psychiatrists. The self-report measures were completed by the participants after the intake interview. All the questionnaires were administered in a totally random order. It took about 30 minutes for all the questionnaires to be completed. No compensation of any sort was offered. All participants signed a written informed consent before the study, and the respective local ethics committees approved the study design.

### 2.4. Statistical Analyses

All analyses were performed using* IBM SPSS for Windows, Version 22.0* [47]. Demographic and clinical data of the participants were analyzed by descriptive statistics. For group comparisons, Student's *t*-test was applied. An exploratory principal components factor analysis was performed, followed by an oblique (direct oblimin) rotation. Factors for extraction were selected by examining eigenvalues [48], and the scree plot, and conducting a parallel analysis [49–52]. The oblique rotation method was chosen because the factors, if any, were theoretically believed to be correlated with each other. To assess the internal consistency of the scale Cronbach's *α* was computed. The correlation coefficient was calculated for test-retest reliability over a 4-week period. For concurrent validity, bivariate Pearson correlation analyses were conducted with the measures of mood severity and negatively biased cognitions. Statistical significance was set at a *p* value of ≤ 0.05.

## 3. Results

### 3.1. Descriptive Statistics

A total of 269 psychiatric outpatients aged 18 and older (61% female; mean age = 36.43 years, SD = 12.55, range = 18–65) were recruited for the study. More than half of the participants (60.6%) were married, and 36.4% of them were single. Almost all of the participants (91.1%) were at least graduates of high school, and 55% of them had a job with a regular income.

The primary diagnoses of the participants were as follows: 51.3% (*n* = 138) depressive disorders (e.g., major depressive disorder, dysthymia) and 46.1% (*n* = 124) anxiety disorders (e.g., generalized anxiety disorder, panic disorder, social anxiety disorder, obsessive-compulsive disorder, and posttraumatic stress disorder). Over one-third of the participants had a comorbid psychiatric diagnosis, the most common being a comorbid depressive and anxiety disorder (37.17%, *n* = 100). Forty-six of the participants (17.1%) reported that they had a family member diagnosed with some kind of mental disorder, and 57 of the participants (21.19%) were also suffering from a comorbid medical condition.

The mean scores and standard deviations of the total score of the CD-Quest and the other scales used in the current study are presented in Table 1.

### 3.2. Internal Consistency

The internal consistency of the CD-Quest was excellent (Cronbach's *α* = 0.93). The corrected item-total correlation (ITC) coefficients ranged from 0.46 (emotional reasoning) to 0.73 (overgeneralization). Deletion of none of the items resulted in an increase in the Cronbach *α* value of the scale. The ITC values are shown in Table 2.

### 3.3. Test-Retest Reliability

For a subgroup of patients (*n* = 50, 18.59%), the test-retest correlation coefficient was calculated over a 4-week period. The results were very satisfactory (*r* = 0.90).

### 3.4. Exploratory Factor Analysis

The Kaiser-Meyer-Olkin measure was 0.93, and Bartlett's test of sphericity was highly significant (*χ* ^2^ = 1964.85, *p* < 0.001). Therefore, the sample size was adequate for a factor analysis. An exploratory principal components factor analysis, followed by an oblique rotation, revealed that a one-factor solution best fit the data. This unidimensional factor structure explained 49.76% of the variance of the scale (eigenvalue = 7.464). Factor loadings of the items are shown in Table 2.

### 3.5. Concurrent Validity

Table 3 presents the correlations between the CD-Quest and other measures used in the study. As hypothesized, the CD-Quest significantly correlated with the CDS as well as the other measures of mood severity and negatively biased thinking (all *p*s < 0.001). As expected, the highest correlation was between the CD-Quest and the total score of the CDS (*r* = 0.75), followed by its interpersonal and achievement subscale scores (*r*s = 0.74 and 0.73, resp.) and the severity of depression (*r* = 0.60). These results demonstrate that there is strong concurrent validity of the scale.

### 3.6. Discriminant Validity

Participants were divided into two groups according to the predefined cut-off scores of the subscales of the HADS, that is, depressed versus nondepressed and anxious versus nonanxious, and the mean scores of the individual items and the total score of the CD-Quest were compared between these groups. Except for the personalizing type of cognitive distortion in depressed individuals, all items were able to distinguish the two groups, suggesting a well discriminating validity of the scale. The results are shown in Table 4.

Each individual item of the scale was further investigated to find out whether any of the cognitive distortions selectively correlated with the severity of depression or anxiety. All individual items were found to be significantly correlated with the HADS subscale scores (all *p*s < 0.01; except for item 4 (emotional reasoning), *p* = 0.025). Except for the magnification/minimization type of distortion, all items were more strongly correlated with the depression score. The results are shown in Table 5.

## 4. Discussion

Cognitive distortions are hypothesized to be central in the development and maintenance of mental disorders according to the theory by Beck [1, 2]. Since there are limited scales which may be utilized to assess cognitive distortions [14–18] and even less translated into Turkish [21], research has been limited on the effect of them to psychopathology and their treatment. The current study aimed to evaluate the psychometric properties of the Turkish version of the CD-Quest, and the results in a psychiatric outpatient sample revealed that the scale had excellent internal consistency, good test-retest reliability, a unidimensional factor structure, and evidence of concurrent and discriminant validity.

The CD-Quest was found to correlate positively with measures of negatively biased cognitions. The scale's association with the CDS was the most highly significant one, demonstrating that the CD-Quest was able to successfully assess the same construct. Moreover, conceptually related measures of negatively biased cognitions have also been found to be moderately to strongly correlated with the CD-Quest adding to the concurrent validity of the scale.

The CD-Quest's significant correlations with both the depression and the anxiety severity may be suggestive of the transdiagnostic feature of cognitive distortions [24], rather than them being specifically more prevalent in depression or anxiety. Although some cognitive distortions may be more frequently encountered in some specific disorders theoretically, for example, catastrophizing in anxiety disorders and discounting the positive in depressive disorders [1–3], the transdiagnostic nature of the construct may actually serve as a guide to a unified approach in treatment. This transdiagnostic nature of the questionnaire has also been reflected in the current study. Since not only the purely depressed and/or purely anxious participants but also the comorbid group scored high on the CD-Quest, it may be hypothesized that the questionnaire is not specific to some specific disorder but is a valid tool to be used transdiagnostically.

The least correlated items with the severity of measures of mood were items 4 (emotional reasoning), 5 (labeling), 10 (personalization), 12 (jumping to conclusions), and 15 (unfair comparisons), all related to anxiety severity. None of the CD-Quest items correlated weakly with the severity of depression. These results suggest that cognitive distortions, albeit transdiagnostic processes, may differentially be activated for specific disorders [1–3]. Further studies should focus on the distinctive characteristics of cognitive distortions in specific disorders and test for their transdiagnostic features in more detail [24].

The unitary factor structure of the CD-Quest is in line with previous reports of measures of cognitive distortions. Both of the Turkish and English versions of the CDS also exhibited unitary factor structures [16, 21]. Since the other measures of cognitive distortions lack published studies on their psychometric properties, and their factor structure in particular, it is not possible to make any comparisons with them.

The current study is the first to provide evidence that the CD-Quest has sound psychometric properties in a Turkish sample. This is also the first published psychometric study of the CD-Quest in a clinical population. The CD-Quest is the second scale available to assess cognitive distortions in Turkish, and with its more comprehensive and briefer structure it is likely that it might provoke more research ideas focusing on the effect of cognitive distortions in the differential diagnosis, development, maintenance, and treatment of mental disorders.

Some limitations of the study may be summarized as follows: (i) limited number of participants, (ii) limited number of psychopathology scales, (iii) low scores on the depression and anxiety rating scales, (iv) focus on only negatively biased cognitions, and (v) use of only self-report measures.

## 5. Conclusions

In conclusion, the current study has provided evidence that the Turkish version of the CD-Quest is a reliable and valid instrument to assess cognitive distortions in a clinical outpatient sample.

## Figures and Tables

**Table 1:** Descriptive statistics of the total score of the CD-Quest, the remaining negatively biased cognition scales, and scales of mood severity.

	Mean	SD	Minimum	Maximum	Possible range
HADS-Dep	7.17	4.75	0	21	0–21
HADS-Anx	9.55	4.84	0	21	0–21
CDS-IP	32.19	12.31	10	67	10–70
CDS-A	31.75	12.56	10	64	10–70
CDS-Total	64.08	24.44	20	128	20–140
ATQ-Total	58.22	26.49	30	150	30–150
DAS-R-NFA	54.82	12.09	13	70	10–70
DAS-R-P	40.12	10.93	11	56	8–56
DAS-R-Total	94.92	21.55	24	102	18–126
CD-Quest Total	20.85	14.85	0	75	0–75

**Table 2:** Factor loadings after the exploratory factor analysis and the corrected item-total correlation of the CD-Quest.

	Factor loading	Communalities	ITC
Item 1	0.761	0.407	0.717
Item 2	0.708	0.480	0.658
Item 3	0.681	0.371	0.623
Item 4	0.514	0.277	0.458
Item 5	0.704	0.446	0.647
Item 6	0.725	0.483	0.672
Item 7	0.718	0.424	0.666
Item 8	0.681	0.336	0.630
Item 9	0.774	0.445	0.733
Item 10	0.729	0.488	0.676
Item 11	0.724	0.440	0.671
Item 12	0.695	0.346	0.639
Item 13	0.712	0.464	0.658
Item 14	0.695	0.347	0.640
Item 15	0.725	0.426	0.668

ITC: corrected item-total correlation.

**Table 3:** Bivariate correlations between the CD-Quest and the other measures.

	*r*
HADS-Dep	0.604^*∗*^
HADS-Anx	0.454^*∗*^
CDS-IP	0.743^*∗*^
CDS-A	0.726^*∗*^
CDS-Total	0.754^*∗*^
ATQ-Total	0.535^*∗*^
DAS-R-NFA	0.303^*∗*^
DAS-R-P	0.387^*∗*^
DAS-R-Total	0.374^*∗*^

^*∗*^ *p* < 0.001.

**Table 4:** Discriminating between clinical and nonclinical participants according to the CD-Quest scores.

CD-Quest	HADS-Dep (Mean ± SD)			HADS-Anx (Mean ± SD)		
	≥7 (*n* = 135)	<7 (*n* = 134)	*t*	≥10 (*n* = 129)	<10 (*n* = 140)	*t*
Item 1	1.66 ± 1.55	0.96 ± 1.11	4.166^*∗*^	1.76 ± 1.49	0.95 ± 1.19	4.893^*∗*^
Item 2	1.93 ± 1.69	0.91 ± 1.29	5.423^*∗*^	2.17 ± 1.69	0.82 ± 1.21	7.487^*∗*^
Item 3	1.56 ± 1.44	0.83 ± 1.04	4.710^*∗*^	1.70 ± 1.45	0.80 ± 1.03	5.872^*∗*^
Item 4	1.80 ± 1.54	1.42 ± 1.26	2.193^*∗∗*^	1.83 ± 1.57	1.44 ± 1.26	2.280^*∗∗*^
Item 5	1.53 ± 1.45	1.13 ± 1.35	2.305^*∗∗*^	1.64 ± 1.51	1.08 ± 1.28	3.257^*∗*^
Item 6	1.76 ± 1.28	0.84 ± 1.05	6.350^*∗*^	1.79 ± 1.34	0.92 ± 1.03	6.018^*∗*^
Item 7	2.01 ± 1.58	1.10 ± 1.17	5.247^*∗*^	2.17 ± 1.59	1.06 ± 1.13	6.605^*∗*^
Item 8	1.99 ± 1.45	1.47 ± 1.31	3.029^*∗*^	2.15 ± 1.42	1.39 ± 1.30	4.543^*∗*^
Item 9	1.53 ± 1.51	0.79 ± 1.00	4.698^*∗*^	1.65 ± 1.50	0.77 ± 1.02	5.649^*∗*^
Item 10	1.01 ± 1.32	0.73 ± 1.10	1.888^*∗∗∗*^	1.22 ± 1.46	0.58 ± 0.86	4.350^*∗*^
Item 11	2.17 ± 1.48	1.40 ± 1.31	4.463^*∗*^	2.36 ± 1.51	1.32 ± 1.21	6.142^*∗*^
Item 12	1.53 ± 1.32	1.14 ± 1.23	2.493^*∗∗*^	1.72 ± 1.46	1.02 ± 1.03	4.503^*∗*^
Item 13	1.62 ± 1.50	0.85 ± 1.21	4.544^*∗*^	1.76 ± 1.64	0.82 ± 1.10	5.664^*∗*^
Item 14	2.08 ± 1.70	1.11 ± 1.20	5.292^*∗*^	2.38 ± 1.71	0.96 ± 1.05	8.222^*∗*^
Item 15	1.37 ± 1.50	0.89 ± 1.23	2.845^*∗*^	1.50 ± 1.60	0.84 ± 1.12	3.915^*∗*^
Total	25.82 ± 15.62	15.65 ± 12.00	5.798^*∗*^	28.24 ± 16.03	14.84 ± 10.55	7.995^*∗*^

^*∗*^ *p* < 0.01, ^*∗∗*^ *p* < 0.05, and ^*∗∗∗*^ *p* = 0.06.

**Table 5:** Correlation between the specific cognitive distortions and measures of mood severity.

	HADS-Dep *r*	HADS-Anx *r*
Item 1	0.404^*∗*^	0.293^*∗*^
Item 2	0.574^*∗*^	0.411^*∗*^
Item 3	0.426^*∗*^	0.316^*∗*^
Item 4	0.259^*∗*^	0.138^*∗∗*^
Item 5	0.292^*∗*^	0.202^*∗*^
Item 6	0.433^*∗*^	0.444^*∗*^
Item 7	0.462^*∗*^	0.398^*∗*^
Item 8	0.411^*∗*^	0.275^*∗*^
Item 9	0.497^*∗*^	0.369^*∗*^
Item 10	0.322^*∗*^	0.190^*∗*^
Item 11	0.447^*∗*^	0.315^*∗*^
Item 12	0.335^*∗*^	0.226^*∗*^
Item 13	0.466^*∗*^	0.376^*∗*^
Item 14	0.557^*∗*^	0.426^*∗*^
Item 15	0.335^*∗*^	0.241^*∗*^

^*∗*^ *p* < 0.01; ^*∗∗*^ *p* < 0.05.
